# Supplementary material for: Social media and public perception as core aspect of public health: The cautionary case of @realdonaldtrump and COVID-19
Source: PLoS One. 2021 May 5;16(5):e0251179. doi: 10.1371/journal.pone.0251179 (PMC8099101; doi:10.1371/journal.pone.0251179)
Supplement: Attachment — Submitted filename: Fuentes and Peterson Response to Reviewers.doc [file pone.0251179.s001.doc]

Siân E Halcrow, Ph.D.
Academic Editor
PLOS ONE	April 20, 2021

Dear Dr. Halcrow,

Please find the responses to each suggestion/comment by the reviewers below and in the track changes version of the text. We were able to fully respond to all comments and make all of the requested changes and additions. Additionally, we formatted the manuscript according to PLOS ONE guidelines and are depositing the raw dataset in Dryad (<https://datadryad.org/stash>) where it will be fully available on publication of the manuscript (if accepted). The ethics statement now appears in the Methods section of the manuscript.

Details of response to reviewers:

Reviewer #1:

Paragraph 3, line 98 is really something that should be in the discussion and not in the intro.

-This sentence was moved to the conclusion.

Why were those specific 7 words chosen. Can you please explain why these were chosen as opposed to pandemic/lockdown/etc?

-This explanation was added Lines 107-112

From May to July 2020 our research group conducted a preliminary examination of six mainstream news media websites, multiple state public health websites and articles/essays by six prominent science writers as initial research for a larger project to examine communication of scientific and public health information during the COVID19 pandemic. Drawing on the preliminary data from May through July we identified seven keywords to sample narratives about the COVID19 pandemic on Twitter.

Has the AIC been previously derived or is this the creation of the authors. Itʼs logical but this is this measure truly valid?

-The AIC is the authors' creation. We developed this metric to assess the relative impact of individual nodes across successively sampled events. This metric is important because given large participant pools, some individuals may miss some events. As such comparing the mean centrality measures of each individual for the events at which they are present is not enough alone. We needed to scale those means to reflect the relative frequency of each node's presence at the successive events. We have not seen a metric like this employed in the SNA literature, and therefore developed it for our data analysis. We added clarifying text to this effect lines 146-152.

Line 166 there is a random “s” there.

-Removed

Line 243 – I would use another word other than “ridiculing”. Although likely his intention its presence is more of an opinion than staying with the facts.

-Changed to “mocking” as this is the term that was used across nearly all journalistic coverage in regard to Trump’s tweets and statements regarding masks and Biden between Sept. 22 and October 2, 2020.

With your questions in the last paragraph, can you suggest some answers/solutions/future directions for research? How can we answer those questions before intervening as suggested.

-Solutions suggested and conclusion revised substantially- see lines 365-385.

Reviewer #2:

The manuscript addresses an important and relevant topic. It is well-done research. However, I would recommend some minor improvements before publication.

-In the manuscript, there should be more relevant references. In total, there are only 26 references including non-academic sources. The COVID-19 pandemic has been quite studied in social science research. Therefore, authors should add some references to strengthen their manuscript.

-All articles suggested by Reviewer #2 were added to the manuscript along with two others.

I would also recommend mentioning research regarding adoption/using Twitter by political actors. In this way, the authors can emphasize the importance of social science research on Twitter.

-This was added to the manuscript along with the citations (see conclusion)

Regarding methods--The methods are well developed. However, the authors should explain why they chose the period from "July 25th, 2020 to November 15th, 2020" and not a different one. Is it somehow connected with the US general election?

-This was clarified with added text lines 121-122: The period of July 25th to November 15th was selected so that the Twitter project data coincided with concurrent elements of the larger project on tracking COVID19 information.

We hope that the revisions are acceptable, and that the manuscript meets with your approval for publication. Please do not hesitate to inform us of any additional revisions as needed.

Sincerely,

Agustín Fuentes

Professor of Anthropology

Princeton University
